# Examining the influence of inflammatory bowel disease medications on sleep quality

**DOI:** 10.1002/jgh3.12871

**Published:** 2023-02-02

**Authors:** Alex Barnes, Paul Spizzo, Peter Bampton, Jane M Andrews, Robert J Fraser, Sutapa Mukherjee, Réme Mountifield

**Affiliations:** ^1^ Department of Gastroenterology Southern Adelaide Local Health Network (SALHN) Flinders Medical Centre Bedford Park South Australia Australia; ^2^ College of Medicine and Public Health Flinders University Bedford Park South Australia Australia; ^3^ Inflammatory Bowel Disease Service, Department of Gastroenterology and Hepatology (CAHLN) Royal Adelaide Hospital Adelaide South Australia Australia; ^4^ School of Medicine, Faculty of Health & Medical Sciences University of Adelaide Adelaide South Australia Australia; ^5^ Department of Respiratory and Sleep Medicine Southern Adelaide Local Health Network (SALHN) Flinders Medical Centre Bedford Park South Australia Australia

**Keywords:** inflammatory bowel disease, methotrexate, obesity, opioids, sleep

## Abstract

**Background and Aim:**

Inflammatory bowel disease (IBD) can disrupt sleep, leading to poor sleep quality. This may in part be due to the symptoms of IBD and the influence of pro‐inflammatory cytokines on sleep. This study aimed to investigate the potential influence of IBD medications on sleep quality.

**Methods:**

An online survey of adults with IBD was conducted, which included measures of sleep quality, IBD activity, anxiety, depression, and physical activity. Logistic regression was used to investigate possible associations between IBD medications (corticosteroids, immunomodulators, biologics, aminosalicyate) and outcome of poor sleep. A generalized linear model was built for outcome of sleep quality score.

**Results:**

There were 544 participants included in the final analysis, median age of 42, and 61% with Crohn's disease. Increased odds of poor sleep were seen in those taking opioids, medications for anxiety or depression, corticosteroids, vitamin D, methotrexate, and infliximab. A multivariate model was built incorporating demographic and IBD variables with opioids present in the final model and associated with increased odds of poor sleep. This was in addition to medications for sleep, depression, anxiety, IBD activity, and body weight. In a multivariate generalized linear model, opioids and methotrexate were associated with worse sleep quality scores.

**Conclusions:**

Opioids were associated with increased odds of poor sleep independent of other factors. This provides further support for avoiding these medications in people with IBD. Infliximab was associated with increased body weight and consequently increased odds of poor sleep.

## Graphical abstract

In a large population of people with Inflammatory bowel disease, poor sleep was more common in those on opioids, infliximab, corticosteroids, vitamin D, and methotrexate. Opioids remained associated with poor sleep when adjusting for other influences of sleep quality including abdominal pain.

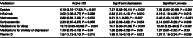

## Introduction

Sleep is an essential biologic function with an important role in overall health. Abnormal sleep has been linked to poor health outcomes including cardiovascular disease,^1^ metabolic syndrome,^2^ and increased all‐cause mortality in some studies,^3^ in addition to significant economic cost in the form of decreased productivity and increased health care utilization.^4^ Sleep has been shown to regulate a number of gastrointestinal functions including gastrointestinal motility and secretion.^5^ Sleep disruption has been associated with increased levels of inflammatory cytokines, such as IL‐6, and TNF‐α, that have been implicated in the pathogenesis of inflammatory bowel disease.^6^, ^7^, ^8^

Inflammatory bowel disease (IBD) is a relapsing–remitting autoimmune disorder that results from a complex interaction between genetics and the environment.^9^ Poor sleep is prevalent in people with IBD with a recent meta‐analysis suggesting a pooled prevalence of 56%.^10^ IBD may impair sleep through its myriad of disabling symptoms, including abdominal pain and nocturnal diarrhea.^11^ Poor sleep is more common in those with IBD than controls,^11^ more common in those with active IBD than inactive IBD,^11^, ^12^ and remains more common in those with inactive IBD than controls.^13^ Endoscopically or histologically active IBD in the absence of any IBD symptoms may be sufficient to disrupt sleep.^14^, ^15^ There have been several association studies of sleep and IBD with comorbid depression^16^, ^17^, ^18^, ^19^, ^20^, ^21^, ^22^, ^23^ frequently associated with poor sleep, and low physical activity associated with poor sleep.^19^, ^24^

The effect of IBD medications on sleep has been investigated with a prospective study following the introduction of a biologic medication with subsequent measurement of subjective sleep quality improving,^20^ likely accompanied by an improvement in IBD activity. Other cross‐sectional studies have been unable to demonstrate a relationship between biologics, immunomodulators, and sleep quality,^14^, ^16^, ^25^ although these studies may have been underpowered. Current use of corticosteroids was associated with worse sleep quality although confounded by IBD activity,^15^, ^23^ however, this was not replicated in other studies.^16^, ^26^, ^27^

Sleep, being an immunologically active state, may be influenced by medications that alter the immune system such as TNF‐a inhibitors.^28^ In people with rheumatoid arthritis,^29^ infliximab, a TNF‐a inhibitor also commonly used in IBD,^30^ was observed to improve some aspects of sleep quality and reduce daytime sleepiness.^31^ Adalimumab, another TNF‐a inhibitor commonly used to treat IBD,^32^ was associated with improved sleep quality in people with psoriasis,^33^ and ankylosing spondylitis.^34^

This study aims to explore the relationship between medications used by people with IBD and sleep quality. It will also consider other influences of sleep quality such as IBD activity, physical activity, and mental health.

## Methods

An online questionnaire was made available to people with IBD via tertiary hospital patient email lists, private gastroenterology practice email lists, and social media. This study received ethics approval from the Southern Adelaide Human Research Ethics Committee (203.20). Individuals with a self‐reported diagnosis of IBD over 18 years of age were invited to participate. Demographic data such as age and sex were recorded, along with data on IBD, which included disease duration and previous surgery. Current medications were recorded including those specifically for IBD, sleep, mental health, and pain control. Medications for sleep were subcategorized as melatonin or, benzodiazepines and zolpidem.

## Questionnaires

### Sleep quality

The Pittsburgh Sleep Quality Index (PSQI) is a validated tool that assesses perceived sleep quality.^35^ The index consists of subscales on sleep duration, sleep disturbance, sleep latency, daytime dysfunction, sleep efficiency, overall sleep quality, and medications for sleep. The score ranges from 0 to 21, with a PSQI >5 considered to represent poor sleep quality.

### IBD disease activity

IBD disease activity was assessed using the Harvey Bradshaw Index in the case of Crohn's disease with HBI >5 considered active disease,^36^ and the Simple Clinical Colitis Activity Index (SCCAI) in the case of ulcerative colitis. An SCCAI >2 was considered active disease.^37^

### Physical activity

Physical activity was assessed using the international physical activity questionnaire short form (IPAQ‐SF).^38^ This allows the calculation of metabolic equivalent of task (MET) values over a one‐week period of walking, moderate and vigorous activity, along with sitting time.

### Anxiety and depression

Anxiety was assessed using the generalized anxiety disorder 7‐item scale (GAD‐7)^39^ with a score over 10 used to indicate clinically significant anxiety. The Patient Health Questionnaire 9 (PHQ‐9) was used to assess depression with a score over 15 used to indicate clinically significant depression.^40^

### Statistical analysis

Statistical analysis was performed using Stata SE 16 (StataCorp, College Station, TX, USA). Inadequate completion of a score or index led to that result not being included. For normally distributed variables, mean and SD were reported, with comparisons made using the Student *t*‐test. For non‐normally distributed variables, median and interquartile range (IQR) were reported, with comparisons made using the Mann–Whitney *U* test. For categorical data, Pearson's *χ* ^2^ test was used or Fisher's exact test when appropriate. Logistic regression was performed for an outcome of poor sleep (PSQI >5). Logistic regression was used to calculate adjusted odds ratios for known IBD activity, anxiety, and depression. A multivariate logistic regression model was built for outcomes of poor sleep including demographic variables. This was model optimized by sequentially adding and removing variables to maximize the likelihood function. A generalized linear model was also constructed for outcome of raw PSQI score with univariate and multivariate regression performed with this optimized by the Bayesian information criterion.

## Results

There were 544 participants who completed the questionnaire. The completion rate for the survey was 93%. Given the method of survey distribution, we are unable to estimate the response rate. The mean age was 42 years (SD 13), 61% had Crohn's disease, and median disease duration was 10 years (IQR 3–17). The mean HBI was 7.2 (3.1) and SCCAI was 7.2 (2.8), with clinically active IBD in 64%. IBD‐related medications included biologics in 54.6% of the cohort, immunomodulators in 37.1%, 5ASA in 35.4%, corticosteroids in 10.1%, and immunomodulator in combination with a biologic in 20.5% (see Table 1).

**Table 1:** Demographics and IBD (inflammatory bowel disease) related data and IBD medications

*n*	544
Age, mean (SD)	42 (13)
Female gender, *n*	436
Weight (kg), mean (SD)	78.9 (20.4)
Height (cm), mean (SD)	167.7 (8.9)
Crohn's disease, *n*	333
Ulcerative colitis, *n*	218
Indeterminate colitis, *n*	15
Disease duration (years, median [IQR])	10 (3–17)
Previous surgery for IBD, *n*	183
Corticosteroids	
Budesonide, *n* (%)	11 (2)
Prednisolone, *n* (%)	44 (8)
Biologics	
Adalimumab, *n* (%)	79 (14)
Infliximab, *n* (%)	95 (17)
Ustekinumab, *n* (%)	67 (12)
Vedolizumab, *n* (%)	50 (9)
Tofacitinib, *n* (%)	6 (1)
Immunomodulator	
Azathioprine, *n* (%)	105 (19)
Mercaptopurine, *n* (%)	53 (10)
Methotrexate, *n* (%)	44 (8)
Aminosalicyate	
Mesalazine, *n* (%)	172 (31)
Sulfasalazine, *n* (%)	21 (4)
Other IBD medication	
Bactrim, *n* (%)	1 (0.01)
Cyclosporine, *n* (%)	0
Tacrolimus, *n* (%)	2 (0.03)
Immunomodulator and biologic, *n* (%)	112 (20)
Aminosalicyate and biologic, *n* (%)	50 (9)
Aminosalicyate, immunomodulator and biologic, *n* (%)	28 (5)
Vitamin D, *n*	152 (28)
Medications for sleep	
Melatonin, *n* (%)	34 (6)
Benzodiazepines or zolpidem, *n* (%)	49 (9)
Medications for depression or anxiety, *n* (%)	128 (23)
Opioids, *n* (%)	78 (14)
Overnight shift work, *n* (%)	31 (6)

### Sleep quality

The mean (SD) PSQI for the cohort was 8.80 (4.56). In reference to different IBD medications (Table 2), the mean PSQI was higher in those on opioids, medication for anxiety or depression, benzodiazepines or zolpidem, melatonin, and corticosteroids (*P* < 0.001 for all). PSQI subscales for medications with a higher PSQI are detailed in Table S1, Supporting information. Corticosteroids were associated with worse sleep efficiency, increased sleep duration, and worse sleep disturbance. Opioids impacted all PSQI subscales apart from need for medications for sleep.

**Table 2:** Pittsburgh sleep quality index (PSQI) scores for inflammatory bowel disease (IBD) medications

Medication	PSQI (mean, 95% CI)	*P* value
Opioids	Yes: 11.92 (11.09–12.75)	<0.0001
	No: 9.05 (8.71–9.40)	
Anti‐anxiety or anti‐depressant	Yes: 10.53 (9.79–11.27)	0.0005
	No: 9.15 (8.78–9.51)	
Medications for sleep	Yes: 11.82 (11.05–12.60)	<0.001
	No: 9.09 (8.75–9.45)	
Benzodiazepines or zolpidem	Yes: 12.06 (11.03–13.09)	<0.0001
	No: 9.21 (8.87–9.56)	
Melatonin	Yes: 11.23 (10.22–12.24)	0.0064
	No: 9.35 (9.01–9.69)	
Vitamin D	Yes: 9.77 (9.16–10.38)	0.26
	No: 9.35 (8.96–9.75)	
5ASA medication	Yes: 9.25 (8.70–9.80)	0.35
	No: 9.58 (9.17–9.99)	
Corticosteroids	Yes: 11.09 (9.97–12.21)	0.0014
	No: 9.29 (8.95–9.64)	
Immunomodulators	Yes: 9.47 (8.90–10.04)	0.99
	No: 9.47 (9.06–8.87)	
Thiopurine	Yes: 9.03 (8.39–9.66)	0.0942
	No: 9.65 (9.26–10.04)	
Methotrexate	Yes: 11.07 (9.84–12.29)	0.0051
	No: 9.33 (8.99–9.68)	
Biologics	Yes: 9.33 (8.89–9.78)	0.38
	No: 9.63 (9.13–10.13)	
Anti‐TNF	Yes: 9.51 (8.92–10.09)	0.88
	No: 9.45 (9.05–9.86)	

Logistic regression was performed for outcome of poor sleep (PSQI >5) (see Table 3), with increased odds of poor sleep seen in those on opioids, medications for sleep including zolpidem and benzodiazepines, medications for anxiety or depression, corticosteroids, vitamin D, methotrexate, and infliximab but not other biologics. No medication was associated with decreased odds of poor sleep. All those on melatonin had poor sleep. Considering PSQI subscales, infliximab was associated with higher sleep disturbance scores and higher scores for needing medications for sleep (see Table S1). Considering a subgroup of those not on any medications for sleep, infliximab remained associated with poor sleep. Methotrexate had higher daytime dysfunction scores, worse sleep efficiency scores, and worse sleep quality scores (see Table S1).

**Table 3:** Table of medications and univariate logistic regression for outcome of poor sleep (Pittsburgh Sleep Quality Index score >5) with odds ratio, 95% confidence interval and *P* value reported

Medication	Poor sleep
Opioids	6.95 (2.49–19.37) *P* < 0.001
Anti‐anxiety or anti‐depressant	1.72 (1.03–2.85) *P* = 0.035
Medications for sleep	13.88 (3.36–57.31) *P* < 0.001
5ASA medication	1.16 (0.77–1.76) *P* = 0.47
Vitamin D	1.98 (1.22–3.23) *P* = 0.006
Corticosteroids	2.69 (1.13–6.45) *P* = 0.026
Immunomodulators	1.28 (0.85–1.92) *P* = 0.23
Methotrexate	3.34 (1.17–9.52) *P* = 0.024
Thiopurine	0.97 (0.63–1.49) *P* = 0.89
Biologics	1.43 (0.95–2.10) *P* = 0.067
Adalimumab	0.85 (0.49–1.46) *P* = 0.56
Infliximab	2.02 (1.11–3.69) *P* = 0.022
Vedolizumab	0.90 (0.047–1.76) *P* = 0.77
Ustekinumab	1.54 (0.80–2.97) *P* = 0.19
Tofacitinib	0.64 (0.12–3.52) *P* = 0.61

All those on melatonin had poor sleep, consequently this was not included.

Combinations of IBD medications were also considered (see Table S2). All of the cohort on opioids and either methotrexate or infliximab had poor sleep. The combination of methotrexate and infliximab did not reach significance for an association with poor sleep (*P* = 0.094). There was no association with poor sleep seen for combinations of aminosalicyates, biologics, and immunomodulators.

### Clinically active IBD

Clinically active IBD was defined as SCCAI >2 or HBI >5, mean SCCAI was 5.7 (4.1), and mean HBI was 5.7 (4.2). Clinically active IBD was associated with poor sleep (see Table S3). Logistic regression was used to calculate adjusted odds ratios by IBD activity for outcomes of poor sleep (see Table 4). Adjusted odd ratios were no longer significant for corticosteroids and methotrexate. Opioids, infliximab, medications for sleep, and vitamin D remained significantly associated with increased odds of poor sleep.

**Table 4:** Logistic regression used to calculate odds ratio for poor sleep adjusted by inflammatory bowel disease (IBD) activity, or depression, or anxiety

Medication	Active IBD	Significant depression	Significant anxiety
Opioids	6.19 (2.19–17.53) *P* = 0.001	7.27 (2.58–20.41) *P* < 0.001	7.33 (2.60–20.63) *P* < 0.001
Infliximab	2.02 (1.08–3.77) *P* = 0.028	2.24 (1.21–4.12) *P* = 0.010	2.19 (1.18–4.06) *P* = 0.013
Methotrexate	2.68 (0.92–7.82) *P* = 0.072	3.21 (1.1–9.28) *P* = 0.027	3.48 (1.20–10.07) *P* = 0.021
Corticosteroids	2.39 (0.97–5.87) *P* = 0.056	2.39 (0.98–5.82) *P* = 0.054	2.14 (0.87–5.26) *P* = 0.096
Medications for sleep	12.13 (2.90–50.69) *P* = 0.001	14.7 (3.55–61.09) *P* < 0.001	13.40 (3.22–55.8) *P* < 0.001
Medications for anxiety or depression	1.29 (0.76–2.19) *P* = 0.34	1.35 (0.80–2.29) *P* = 0.25	1.37 (0.81–2.32) *P* = 0.23
Vitamin D	1.87 (1.13–3.10) *P* = 0.015	1.89 (1.15–3.10) *P* = 0.012	1.97 (1.19–3.24) *P* = 0.008

### Depression

Clinically significant depression (PHQ‐9 >15) was associated with poor sleep, (see Table S3). Logistic regression was used to calculate adjusted odds ratios by depression (PHQ‐9 >15) for outcomes of poor sleep (see Table 4). After adjustment, corticosteroids were no longer significant (*P* = 0.054), and other medications remained significantly associated with poor sleep.

### Anxiety

Clinically significant anxiety (GAD‐7 >10) was associated with poor sleep (see Table S3). Logistic regression was used to calculate adjusted odds ratios by anxiety (GAD‐7 >10) for outcomes of poor sleep (see Table 4). After adjustment, corticosteroids were no longer significant (*P* = 0.096), and other medications remained significantly associated with poor sleep.

### Physical activity

Physical activity as measured by total METs, sitting time, and vigorous METs was not associated with any sleep quality measure. Further analysis was consequently not undertaken.

### Multivariate regression

A multivariate model was constructed for outcome of poor sleep that included medications—opioids, medications for sleep, Infliximab, and vitamin D. The model also included demographic variables and IBD‐related variables such as disease duration (for univariate logistic regression see Table S4). In the final model (see Table 5), opioid usage and medications for sleep remained associated with increased odds of poor sleep. Infliximab and vitamin D were not included in the final model. Other variables in the final model included body weight, IBD disease duration, clinically significant anxiety, clinically significant depression and clinically active IBD.

**Table 5:** Final multivariate logistic regression model for outcome of poor sleep including opioids, infliximab, vitamin D, medications for sleep, and demographic variables

Variable	Odds ratio	Confidence interval	*P* value
Opioids	3.08	1.04–9.122	0.041
Benzodiazepines or zolpidem	9.21	2.08–40.86	0.003
Weight	1.02	1.01–1.03	<0.001
IBD disease duration	1.02	1.00–1.04	0.042
Clinically significant anxiety	3.82	1.88–7.78	<0.001
Clinically significant depression	3.67	1.21–11.08	0.021
Active IBD	2.56	1.64–4.00	<0.001

IBD, inflammatory bowel disease.

Infliximab was not significantly associated with poor sleep when adjusted for by body weight (see Table S5). People on infliximab had a higher body weight than the remainder of the cohort (79.56 *vs* 72.45, *P* = 0.024). Infliximab remained significantly associated with poor sleep when adjusted by other variables in the final model excluding body weight (Table S5). This was similarly observed with vitamin D with those on vitamin D having a higher body weight than the remainder of the cohort (80.43 *vs* 71.19, *P* = 0.0005). Vitamin D remained significantly associated with poor sleep when adjusted by the other variables in the final model excluding body weight (see Table S5). Sub scores from IBD clinical activity were considered for abdominal pain and nocturnal diarrhea. Opioids were associated with abdominal pain (*P* < 0.001) but not nocturnal diarrhea (*P* = 0.19). Adjusted odds ratio for poor sleep for those on opioids remained significant after adjustment for abdominal pain. Opioids were associated with longer IBD disease duration (14.6 [11.8–17.5] *vs* 11.5 [10.6–12.4], *P* = 0.014), and higher SCCAI scores (*P* < 0.012) or HBI scores (*P* < 0.0001).

A generalized linear model was constructed for outcome of PSQI score with univariate (see Table S6) and multivariate regression performed (see Table S7). With respect to IBD medications, the univariate regression was again significant for methotrexate and corticosteroids with increased odds of poor sleep but not infliximab and vitamin D. The final multivariate model included methotrexate in addition to opioids and medications for sleep including melatonin.

## Discussion

Here we have described the results of an online questionnaire demonstrating a relationship between IBD medications and sleep quality in people with IBD. Opioids, commonly prescribed in IBD, were associated with increased odds of poor sleep as part of a multivariate model including clinically active IBD, body weight, depression, anxiety, IBD disease duration, and medications for sleep. Infliximab and vitamin D were associated with poor sleep, but this appeared to be confounded by body weight, with both medications not included in the final multivariate model. Methotrexate was associated with higher PSQI scores. This study builds on previous work that did not show any significant relationship between sleep quality and biologics or immunomodulators.^25^

Chronic opioid usage in people with IBD has been associated with increased all‐cause mortality,^41^, ^42^ worse IBD outcomes such as infection,^43^ worse quality of life,^44^ and increased health care utilization.^45^ In our population, opioids were associated with worse sleep quality. Opioids are known to alter sleep architecture^46^ and are associated with sleep disordered breathing,^47^ in particular central sleep apnoea.^48^ We also note that opioids may be a marker of more severe IBD.^49^ In our study, opioids were associated with longer disease duration and higher clinical disease activity scores. Opioids remained associated with poor sleep following adjustment for abdominal pain; however, it is possible that other types of pain contributed to sleep quality that was not accounted for.

Infliximab has been associated with weight gain in people with IBD,^50^, ^51^, ^52^, ^53^, ^54^, ^55^ with the suggestion that infliximab may inhibit leptin production.^56^ Infliximab‐related weight gain has also been observed in cohorts of people with rheumatoid arthritis^57^, ^58^, ^59^ and psoriasis.^60^, ^61^, ^62^ Vitamin D deficiency has been associated with obesity,^63^, ^64^ although to the authors' knowledge there is no known association between vitamin D replacement and weight gain.^65^ Increased body weight is a risk factor for sleep apnoea^66^ and perhaps the associated weight gain from infliximab or vitamin D deficiency increases the likelihood of sleep apnea and consequently more likely to have poor sleep. Vitamin D deficiency has been associated with increased risk of sleep disorders^67^ and supplementation has been associated with improvement in sleep quality.^68^

Corticosteroids, known to cause sleep disturbances,^69^ were associated with worse sleep quality scores and poor sleep. This replicates previous work showing worse sleep quality scores^15^, ^23^ in those on corticosteroids. The association with poor sleep was confounded by firstly IBD activity and also mental health scores, of which corticosteroids are well known to influence.^70^

Methotrexate was associated with higher PSQI scores on multivariate regression but not increased odds of poor sleep on multivariate regression. This may be due to the small number of participants on methotrexate (8%) and consequent vulnerability to some yet unidentified bias. Methotrexate is associated with fatigue,^71^, ^72^ which commonly limits its use. Associations studies in a rheumatoid arthritis population have not demonstrated a relationship between sleep quality and methotrexate;^73^ however, in other prospective studies, introduction of methotrexate did not lead to any improvement in sleep quality—unlike introduction of TNF‐a inhibitors.^74^, ^75^

Limitations of this study include selection bias as a result of the use of an online questionnaire that may attract people with sleep problems. The rate of poor sleep in this cohort (75%) was higher than that reported in a recent meta‐analysis on the prevalence of poor sleep in IBD^10^ (56%), although a number of other studies have reported higher rates of poor sleep^15^, ^21^, ^25^, ^76^, ^77^ than seen in our cohort. Similarly, the form of survey and method of recruitment is likely responsible for the predominantly female cohort. Reporting bias may also be significant, noting a study of people with Crohn's disease reported worse sleep quality than that observed by objective measures.^26^ The absence of objective measures of sleep quality and objective IBD activity is also considered a limitation. Further studies should consider objective measures of IBD activity and sleep quality. Studies incorporating mental health interventions in those with poor sleep should be pursued. Consideration should also be given to examining the relationship between serum levels of vitamin D and sleep quality in an IBD population.

## Conclusions

A large survey of people with IBD has shown that opioids are associated with increased odds of poor sleep. Infliximab and vitamin D usage was associated with poor sleep; however, this was confounded by higher body weight and consequently perhaps increased rates of sleep apnea. Further studies are required to confirm these results and should incorporate objective measures of sleep quality and IBD activity.

## Supporting information


**Appendix S1.** Supplementary Information.

## Data Availability

The data underlying this article are available upon request to the author.
