# Supplementary material for: Examining the influence of inflammatory bowel disease medications on sleep quality
Source: JGH Open. 2023 Feb 2;7(3):190–6. doi: 10.1002/jgh3.12871 (PMC10037038; doi:10.1002/jgh3.12871)
Supplement: Supplementary file 1 — Appendix S1. Supplementary Information. [file JGH3-7-190-s001.docx]

Supplementary table 1: Pittsburgh sleep quality index components for medications – opioids, corticosteroids, and vitamin D. Arrows to indicate significantly higher or lower score compared to remainder of cohort (*p=0.05, ** indicates p<0.005, *** indicates p<0.0005).
Sleep efficiency is the time in bed that you are asleep divided by total time in bed. Sleep latency is the time taken to fall asleep.

| PSQI component | Opioids | Corticosteroids | Medication for anxiety or depression | Infliximab | Methotrexate | Vitamin D | Medications for sleep |
| --- | --- | --- | --- | --- | --- | --- | --- |
| Sleep duration | ↑*** | ↑** | ↔ | ↔ | ↔ | ↔ | ↔ |
| Sleep disturbance | ↑*** | ↑** | ↑*** | ↑** | ↑ | ↑*** | ↑** |
| Sleep latency | ↑*** | ↑* | ↑** | ↔ | ↔ | ↔ | ↑*** |
| Daytime dysfunction | ↑*** | ↑* | ↑** | ↔ | ↑** | ↑** | ↑*** |
| Sleep efficiency | ↑*** | ↑*** | ↑*** | ↔ | ↑*** | ↔ | ↑** |
| Sleep quality | ↑*** | ↑* | ↑* | ↔ | ↑** | ↔ | ↔ |
| Medications for sleep | ↔ | ↔ | ↔ | ↑** | ↔ | ↔ | ↑** |

Supplementary table 2: Univariate logistic regression for poor sleep (PSQI > 5) for IBD maintenance medication combinations. All of the cohort on opioids in addition to either methotrexate or infliximab had poor sleep, and consequently these combination medication variables were not included in the table.

|  | Odds ratio, 95% confidence interval, p value |
| --- | --- |
| Opioids and biologics | 1.50 (1.15-1.97) p=0.003 |
| Methotrexate and infliximab | 5.64 (0.74-.42.76) p=0.094 |
| Immunomodulator and biologic | 1.39 (0.83-2.32) p=0.20 |
| Aminosalicyate and Immunomodulator | 0.89 (0.49-1.61) p=0.71 |
| Aminosalicyate and biologic | 0.73 (0.38-1.37) p=0.33 |
| Aminosalicyate and immunomodulator and biologic | 0.79 (0.34-1.84) p=0.59 |
| Biologic without any other IBD maintenance medication | 1.27 (0.81-2.00) p=0.29 |
| Immunomodulator without any other IBD maintenance medication | 0.98 (0.50-1.95) p=0.97 |
| Aminosalicyate without any other IBD maintenance medication | 1.38 (0.78-2.44) p=0.26 |

Supplementary table 3: Sleep quality by clinically active IBD (SCCAI > 2 or HBI > 5), clinically significant depression (PHQ9 > 15), clinically significant anxiety (GAD7 > 10) with differences in sleep quality and odds ratios for poor sleep (PSQI > 5) and clinically significant insomnia (ISI > 14).

|  | Poor sleep  (Odds ratio, 95% CI) |
| --- | --- |
| Clinically active IBD | 4.16 (2.79-6.21) p<0.001 |
| Clinically significant depression | 9.72 (3.51-26.97) p < 0.001 |
| Clinically significant anxiety | 6.53 (3.42-12.46) p < 0.001 |

Supplementary table 4: Univariate logistic regression for poor sleep (PSQI > 5) for demographic.

|  | Odds ratio, 95% confidence interval, p value |
| --- | --- |
| Crohn’s disease | 1.75 (1.19-2.57) p=0.004 |
| IBD disease duration | 1.04 (1.01-1.06) p<0.001 |
| Previous surgery for IBD | 1.54 (1.00-2.37) p=0.049 |
| Overnight shift work | 1.28 (0.48-3.43) p=0.61 |
| Current smoker | 1.71 (0.69-4.19) p=0.24 |
| Current alcohol usage | 1.17 (0.77-1.77) p=0.47 |
| Body mass index | 1.07 (1.03-1.11) p < 0.001 |
| Weight | 1.02 (1.02-1.03) p<0.001 |
| Height | 1.01 (1.00-1.01) p<0.001 |
| Male gender | 0.81 (0.50-1.30) p=0.38 |
| Age | 1.02 (1.01-1.03) p=0.001 |

Supplementary table 5: Adjusted odds ratios for outcome of poor sleep for infliximab and vitamin D by variables included in the final multivariate model. Note – odds ratios adjusted by anxiety, depression and IBD activity have been described in table 4.

| Variable | Opioids | Medications for sleep | Weight | IBD disease duration |
| --- | --- | --- | --- | --- |
| Infliximab | 2.02 (1.09-3.71) p=0.024 | 2.00 (1.09-3.70) p=0.025 | 1.72 (0.93-3.18) p=0.085 | 1.99 (1.08-3.66) p=0.026 |
| Vitamin D | 1.82 (1.12-2.99) p=0.016 | 1.88 (1.15-3.09) p=0.012 | 1.62 (0.98-2.68) p=0.058 | 1.85 (1.13-3.03) p=0.014 |

Supplementary table 6: Generalised univariate linear regression with outcome of Pittsburgh Sleep Quality Index score (PSQI score).

|  | Exponential of coefficient, 95% CI, p value |
| --- | --- |
| Crohn’s disease | 1.35 (0.68-2.65) p=0.39 |
| IBD disease duration | 1.02 (0.99-1.05) p=0.25 |
| Previous surgery for IBD | 1.51 (0.75-3.03) p=0.25 |
| Overnight shift work | 0.85 (0.21-3.51) p=0.82 |
| Current smoker | 2.46 (0.64-9.36) p=0.19 |
| Current alcohol usage | **0.36 (0.18-0.72) p=0.004** |
| Obesity | **0.26 (2.16-8.39) p<0.001** |
| Weight | **1.03 (1.01-1.04) p < 0.001** |
| Height | 1.00 (0.99-1.01) p=0.80 |
| Male gender | *0.48 (0.21-1.10) p=0.083* |
| Age | 1.00 (0.99-1.00) p=0.51 |
| Opioids | **17.54 (7.09-43.39) p<0.001** |
| Anti-anxiety or anti-depressant | **3.98 (1.84-8.60) p<0.001** |
| Benzodiazepines or zolpidem | **21.77 (6.35-74.58) p<0.001** |
| Melatonin | **6.56 (1.70-25.24) p=0.006** |
| 5ASA medication | 0.72 (0.36-1.44) p=0.35 |
| Vitamin D | 1.52 (0.73-3.17) p=0.26 |
| Corticosteroids | **6.04 (2.01-18.09) p = 0.001** |
| Immunomodulators | 0.99 (0.50-1.97) p=0.99 |
| Methotrexate | **5.67 (1.69-18.98) p=0.005** |
| Thiopurine | 0.54 (0.26-1.11) p=0.094 |
| Biologics | 0.74 (0.38-1.44) p=0.39 |
| Adalimumab | 0.81 (0.32-2.09) p=0.67 |
| Infliximab | 1.28 (0.54-3.07) p=0.57 |
| Vedolizumab | 0.3 (0.12-1.14) p=0.082 |
| Ustekinumab | 1.01 (.037-2.77) p=0.98 |
| Tofacitinib | 2.82 (0.12-65.47) p=0.52 |
| Opioids and biologics | **1.55 (1.06-2.28) p=0.025** |
| Methotrexate and infliximab | 6.85 (1.10-42.67) p=0.039 |
| Immunomodulator and biologic | 0.92 (0.41-2.07) p=0.84 |
| Aminosalicyate and Immunomodulator | 0.57 (0.21-1.59) p=0.29 |
| Aminosalicyate and biologic | 0.49 (0.16-1.51) p=0.21 |

Supplementary table 7: Generalised multivariate linear regression with outcome of Pittsburgh Sleep Quality Index score (PSQI score), optimised by Bayesian information criterion.

|  | Exponential of cofficient, 95% CI, p value |
| --- | --- |
| Opioids | 6.83 (2.79-16.69) p<0.001 |
| Melatonin | 5.52 (1.54-19.78) p=0.009 |
| Benzodiazepines or zolpidem | 12.23 (4.02-37.16) p<0.001 |
| Methotrexate | 5.99 (2.01-17.85) p=0.001 |
| Clinically significant depression | 9.62 (4.10-22.54) p<0.001 |
| Clinically significant anxiety | 4.53 (2.21-9.28) p<0.001 |
| Clinically active IBD | 2.45 (1.29-4.62) p = 0.006 |
